# Bringing Trust Building to Life Within Health Policy-Making: Comment on "Placing Trust at the Heart of Health Policy and Systems"

**DOI:** 10.34172/ijhpm.8662

**Published:** 2024-09-15

**Authors:** Felix Gille, Paola Daniore, Federica Zavattaro

**Affiliations:** ^1^Digital Society Initiative, University of Zurich, Zürich, Switzerland.; ^2^Institute for Implementation Science in Health Care, University of Zurich, Zürich, Switzerland.

**Keywords:** Trust, Policy, Performance, Governance, Trustworthiness

## Abstract

Public trust is crucial for health policy-making as it is closely linked to public participation and policy-makers’ legitimacy. While we have witnessed increasing interest in issues of public trust, especially since COVID-19, we argue that efforts should be made to deeply integrate the concept of public trust into health policy-making from the design to the implementation, assessment, and evaluation processes across the public health and medical domains. We propose four key aspects to consider for building public trust in the healthcare system, which have emerged from our past and ongoing health policy research on public trust: to understand trust before aiming to build trust; to provide tailored guidance on trust building in health policy; to develop trust performance indicators to assess trust interventions; and to implement targeted communication strategies.

## Introduction

 Public trust is instrumental for efficient healthcare and successful health system governance. When the public trusts health system actors, the public is more likely to participate in health system activities such as vaccination campaigns, organ donation, digital health interventions and public health measures to fight diseases. As “trust is a bet about the future contingent actions of others”^1^ (p. 25), the public partakes in these health system activities in anticipation of a future net-benefit for the individual, the public and the health system. Additionally, public trust is an important component of health system legitimacy, as trusted health system actors hold the public’s legitimacy to act in the public’s best interest.^2^ In contrast to a private person and professional, we understand the public to be the general community. Considering the critical role of public trust in the healthcare system, McKee and colleagues asked “[…] do health systems prioritise building and maintaining trust?”^3^ (p. 1).

 We witness considerable interest in trust building and trustworthiness in the broad digital health domain, where trust is often understood to be built by privacy protection, data security and personal autonomy. Trust is as a critical facilitator for acceptance and implementation of digital health interventions.^4^ Looking at the history of trust research, two prominent research areas on trust in the medical field are first, the patient-doctor relationship, where trust is linked to exchange of information and adherence to treatment plans; and second, vaccine hesitancy, where lower levels of trust in vaccination programs are linked to concerns about vaccine safety and lower trust in healthcare providers, as well as the general system.^5,6^ However, when considering the overall health system, trust remains a topical issue only at the margins of health policy-making activities over the recent years. Certainly, the COVID-19 pandemic and the introduction of artificial intelligence in medicine moved trust into the spotlight.^2^ Yet, it remains an open question in how far we are able to keep the momentum and incorporate trust as an integral component of health system policy and healthcare administration on a larger scale. If we can establish trust as a quality standard and performance indicator for successful healthcare systems, we might reach the point where principles of trust building are embedded by default in the design, implementation, assessment, and evaluation process of health policies across the public health and medical domains.

 To advance the integration of trust elements into health policy-making and healthcare administration, we contribute to McKee’s and colleagues’ timely editorial by proposing four aspects that are key for building public trust in the healthcare system: conceptual precision; tailored guidance; trust performance indicators; and communication. The four elements emerged as significant in our past and ongoing health policy research on public trust in the health system.^2^

## Conceptual Precision: What Are We Talking About?

 McKee and colleagues, rightly so, discuss the often-highlighted conceptual complexity and missing common understanding of trust. The relational construct of trust is context-specific and perceptions of trustworthiness of involved actors are tied to early childhood, positive or negative trust experiences during lifetime, and cultural norms and values of the society.^7,8^ Importantly, the relationship between trustworthiness and trust is complex, with no direct causality linking the two. Nevertheless, we can derive a set of central themes in trust building that are common to most trust descriptions: (*a*) trust is a relational, future-oriented concept where the trusted party acts in the interest of the trusting party to reach a beneficial outcome for the trusting or both parties; (*b*) the act of trusting carries the risk of being misused, resulting in adverse outcomes; (*c*) to build trust the trusting party needs information about the party seeking trust, and it is up to the trusting party to determine trustworthiness, subsequently leading to the establishment of trust; (*d*) trust cannot be guaranteed, which means that investing a considerable amount of resources into trust building does not always result in a successful trust relationship.^2,9^

 To make trust a workable and meaningful concept, it is important for researchers to report with precision the trust concept under study, and for policy-makers, it is critical to invest in comprehensively understanding trust within the focus area of their health policy. Details matter in trust building.

## Tailored Guidance: What Should We Do?

 After understanding what trust is, we ask: What actionable steps can we take to advance trust building efforts?

 Following the commonly used phrase “Trust is difficult to win and easy to lose,” it is advisable to balance proactive and reactive approaches to trust building. Reactive measures should follow an incident, such as a health system data leak or misuse of health data. However, reactive measures for reestablishing trust are complicated to implement and resource intensive. A proactive approach with preventive measures is needed to build trust and prevent its potential loss. Proactive approaches involve a continuous and prolonged process that follows deliberate, tailored, and actionable interventions by actors aiming to build trust. Clear leadership and commitment are necessary to drive the trust building process. Within private and public organizations, early evidence suggests that trust building needs to be driven by executives committing resources to the process and acting as role models for a trust culture. While there is no guarantee that trust will be established, taking intentional and evidence-informed steps increase the likelihood of trust building. Interviews in ongoing studies on the role of trust in the European and national health data sharing policy processes with health policy-makers suggest that, while they recognize the importance of public trust, particularly in initiatives like the European Health Data Space due to the sensitivity of health data, they lack concrete insights on how to build trust. This often results in an unstructured approach to trust building. There is a consensus that useful guidance on trust building in health policy is lacking. Policy-makers often turn to no-brainer concepts, such as privacy and data protection, relying on existing legislation as a means of ensuring trustworthiness. Alternatively, they may rely on “gut feelings” to approach trust. Professionals ask for tailored guidance and context-specific solutions that fit a data flow, such as trustworthy patient data management systems for secondary data use. Similar findings seem to emerge in an ongoing study on the role of trust in the digital economy in Switzerland.

 Depending on the stage of the policy process, policy-makers sometimes seem to delegate trust building to colleagues or institutions closer to the public, missing the opportunity to establish trust from the outset. Public trust should be in focus during the entire health policy process from the start to the end. Especially so as legal acts and policies are understood as vital instruments to build trust. The existence of robust polices and laws as well as the rule of law is for large parts of the public a foundation for a trustworthy health system. Policy-makers can actively contribute to trust building by explicitly signalling public trust in the legislation itself and setting public trust as one of the policy outcomes to be achieved in the implementation phase. To do so, practical and context-specific guidance on trust building in health policy is needed. We need to ensure that critical trust building aspects are explicitly mentioned in the policies and very importantly applied in practice by those implementing the policies.

## Trust Performance Indicators: How Do We Assess Success?

 We can develop different methods to assess evidence about levels of trust. Taylor and colleagues call for methodological creativity in measuring trust.^10^ We need to go beyond conventional survey designs and consider a methodologically rich and holistic approach to assessing trust. One-off data collection, such as household-surveys or surveys administrated during a study, provide an informative snapshot of public perceptions of trust. However, for many health system activities and health policy processes, more dynamic and more tightly knit methods are needed to gather routine data about trust. The collected evidence will proactively support the policy process and health system intervention itself. The idea is to accompany the design, implementation, monitoring and evaluation process of health policies and system activities throughout their full lifecycle.

 One possible way forward is the introduction of Trust Performance Indicators, similar to existing health and quality performance indicators used in many health systems for quality and performance control.^11,12^ Following a precise conceptual understanding of trust which is derived from (*a*) a well formulated definition of trust and (*b*) a robust conceptual framework of trust, such indicators can be implemented to routinely collect the necessary data about health system activities’ trustworthiness. For example, we know that data security contributes to the perception of trustworthy health data sharing activities. To inform the levels of trust associated with data security, one could collect data about data breaches, lost data, and hacking. As several relevant indicators already exist as for example in the context of Digital Trust the Digital Intelligence Index, rather than reinventing the wheel, a better approach could entail re-arranging existing indicators and introducing new ones to ensure comprehensive coverage of a conceptual framework of trust.^13^ A potential challenge with indicators is that they may lead to a focus on meeting targets, causing employees to shift their attention away from the actual work. Further, dynamic and complex trust relationships will make it challenging to define indicators that are meaningful over a prolonged period. Last, the overall number of indicators used in a specific setting needs to balance between usability and coverage. Few indicators will provide a superficial or fragmented picture of trust, while lots of indicators will overburden the work process, potentially leading to poor data about trustworthiness or low acceptance of the indicators. Overall, an important, yet often overlooked, contribution such indicators can make is to provide the necessary evidence to inform economic evaluation and resource allocation in trust building, particularly as health systems are faced with scarce resources. While trust is commonly understood as instrumental, resources to build trust are not endless. Therefore, evidence-informed resource allocation is crucial in trust building.

## Communication: Tell the Truth!

 Without the exchange of truthful information, it will not be possible to establish trust.^14^ Intentionally communicating untruthful information carries a high risk of fundamentally undermining public trust. To be able to assess trustworthiness of a new health system activity, such as the ongoing introduction of national electronic health record systems in several European countries, we consider information shared with us by others, as well as past experiences with comparable health system activities.^15^ We combine this information with perceptions about the present capabilities of the health system to fulfil what we are ought to trust it for, and future anticipations about the potential beneficial outcome of the trusted health system activity. The need of trust building communication to cover threefold information relating to the past, present and future requires well-developed public information campaigns. It is essential that both the sender of information and the information itself are understood by the receiver as truthful. This admittingly presents a crux, given that truthful information competes against untruthful information in the public sphere. It is increasingly difficult for lay people to separate truth from untruth in infodemics. Possible remedies to this problem are certification processes that certify the trustworthiness of actors, as well as efforts to increase public health literacy to enable the public to understand and assess complex health system activities and to derive conclusions about their trustworthiness. Communication campaigns need to not only use targeted language for different audiences, but also employ relatable, understandable, and competent spokespersons explaining the benefits to the public of health system activities and addressing public concerns with easy-to-understand narratives. The use of relatable examples to explain to the public the anticipated direct benefit of health system activities is likely to catch more attention and to convey important information compared to the more commonly-used technical medical jargon.^2^

## Conclusion

 We fully agree with McKee and colleagues, that trust is instrumental for health system performance, state legitimacy and public participation in health system initiatives. Without appropriate levels of public trust, health system initiatives are at risk of failing. It is imperative to first understand the concept of trust when talking about trust and to embed trust building principles in health policies from the get-go. Tailored guidance on trust building in health policy is needed to enable policy-makers to actively contribute to trust building through policy-making. A comprehensive trust monitoring system making use of trust performance indicators has the ability to provide us with timely real-world evidence about trust in health system interventions and their underlying policies throughout the design, implementation, monitoring and evaluation phases. Lastly, and most importantly, understandable and targeted communication is the lifeblood of trust building.

## Acknowledgement

 The section about trust performance indicators builds on “We need to discuss the economics of trust building in digital health,” FG co-authored with Laura Maaß, Benjamin Ho, and Divya Srivastava, which is currently under review.

## Ethical issues

 Not applicable.

## Conflicts of interest

 FG receives funding from Novartis AG, Stiftung Sanitas Krankenversicherung, Schweizerische Akademie der Technischen Wissenschaften, World Health Organization (WHO) outside of the submitted work.

## Disclaimers

 We reflect upon unpublished preliminary findings of our ongoing studies: (A) Digital Trust, funded by the Swiss Academy of Engineering Sciences; (B) Public trust in a Swiss health data space, funded by Novartis AG and Stiftung Sanitas Krankenversicherung; (C) The role of trust in the European and national health data sharing policy process: from Brussels to Bern, Rome, and Paris, funded by the Digital Society Initiative (DSI), University of Zurich; (D) Data handling, security and protection: reaching conceptual equivalence for the concept of public trust in national electronic health records in Switzerland and neighbouring countries, funded by the DSI, University of Zurich.
